# Uncertainty analysis of species distribution models

**DOI:** 10.1371/journal.pone.0214190

**Published:** 2019-05-23

**Authors:** Xi Chen, Nedialko B. Dimitrov, Lauren Ancel Meyers

**Affiliations:** 1 Graduate Program in Operations Research Industrial Engineering, The University of Texas at Austin, Austin, Texas, United States of America; 2 Department of Integrative Biology, The University of Texas at Austin, Austin, Texas, United States of America; 3 The Santa Fe Institute, Santa Fe, New Mexico, United States of America; University of Pittsburgh Graduate School of Public Health, UNITED STATES

## Abstract

The maximum entropy model, a commonly used species distribution model (SDM) normally combines observations of the species occurrence with environmental information to predict the geographic distributions of animal or plant species. However, it only produces point estimates for the probability of species existence. To understand the uncertainty of the point estimates, we analytically derived the variance of the outputs of the maximum entropy model from the variance of the input. We applied the analytic method to obtain the standard deviation of dengue importation probability and Aedes aegypti suitability. Dengue occurrence data and Aedes aegypti mosquito abundance data, combined with demographic and environmental data, were applied to obtain point estimates and the corresponding variance. To address the issue of not having the true distributions for comparison, we compared and contrasted the performance of the analytical expression with the bootstrap method and Poisson point process model which proved of equivalence of maximum entropy model with the assumption of independent point locations. Both Dengue importation probability and Aedes aegypti mosquito suitability examples show that the methods generate comparatively the same results and the analytic method we introduced is dramatically faster than the bootstrap method and directly apply to maximum entropy model.

## Introduction

Species distribution models [1–3] are commonly used to predict the geographic distributions of animals or plants species. They are applied in species conservation [4], ecology [5], and other fields. Some SDMs, like the maximum entropy model, are used to predict the probability for the species being present. Others, like Poisson point process models, are used to model the intensity of the species per unit area.

Quantifying the uncertainty of maximum entropy models can help biologists allocate sampling efforts more efficiently. For places with the same probability estimate, different uncertainty estimates can help differentiate the need for further sampling effort. It may be possible to lower uncertainties in the estimates by choosing sampling locations carefully. However, the independence between sample units need to be guaranteed to maintain the independence assumptions underlying a maximum entropy model. Quantifying the uncertainty also helps biologists have an idea of the amount of data sufficient to estimate probabilities across the geographic area. Knowledge of uncertainty can help answer questions such as: What is the benefit of collecting an additional 1000 presence only data points? What are low and high scenarios for the output estimates?

Unfortunately, most SDM methodology focuses on using point estimates. Point estimation involves using a single value for estimating target population parameters from sample data. However, the estimations are usually not equal to the target population parameters exactly, and so the accuracy of the estimations is important. A well accepted method of describing the uncertainty of the estimations is to look at their variance. With the variance of the estimates, one can compute confidence intervals, an interval that contains the true parameter with a certain confidence [6]. With current practice, SDMs only produce point estimates for the predicted probability or intensity at all species locations and background points without any corresponding uncertainty estimates at these locations.

To address this lack of uncertainty quantification in SDM, one must refer both to the SDM methodology and statistical methodology in quantifying uncertainty of point estimates. One of the most popular methods for SDM is the maximum entropy model. The conventional maximum entropy model was first formulated by Jaynes in 1957 [7] based on Shannon’s measure of entropy [8] (see details in [9]). MAXENT incorporating the effect of actual occurrence data, became popular among biologists in modeling species distribution with the contribution of MaxEnt software [1, 10, 11]. The mathematical equivalence of MAXENT, model used in MaxEnt software, and Poisson point process models (Poisson PPMS has been shown in [12]. Poisson PPMS may be fitted in the ‘spatstat’ package in R, which provides a way of assessing model uncertainty by providing standard error estimates [13]. To quantify uncertainty in point estimates, bootstrap methods are popular. Bootstrap uses computer-intensive simulation to calculate standard deviations of the estimated parameters, and is broadly applied in the biology field [14–17]. In this paper, we adopt the maximum entropy method and compare the analytical expression of the standard deviation with the standard deviation calculated through bootstrap method and Poisson point process model (PPM) approach.

In this article we consider quantifying the uncertainty in SDM. We focus specifically on the maximum entropy SDM methodology. A significant reason for the popularity of the maximum entropy methodology is its applicability to presence-only data with least assumptions [18]. For traditional statistical estimation methods like regression, both of the presence and absence of the species are required. However, in real cases, biologists often only know the places a species has been observed, while lacking information about absences of species.

Our main contribution is analytically deriving an expression of the standard deviation of the target species distribution probabilities and comparing the results with bootstrap methods and standard deviation calculated through Poisson PPM approach. We show that the three methods generate comparatively the same results and our analytic model uncertainty calculation procedure is dramatically faster than the boostrap method and more proper comparing to Poisson PPM without independence assumption and provided a direct result to maximum entropy model.

## Materials and methods

### Maximum entropy model

Consider a region with geographic divisions given by *X* = {*x*_1_, *x*_2_, …, *x*_*n*_}. Suppose some species lives in the region, and the fraction of the species that lives in division *i* is *p*_*i*_. A basic goal in SDM is to reconstruct the geographic distribution *P* = {*p*_1_, *p*_2_, …, *p*_*n*_}. To do this, we have some species occurrence data *O* = {*o*_1_, *o*_2_, …, *o*_*n*_}, where each *o*_*i*_ specifies the number of times the species has occurred in division *i*. The occurrence data can be viewed as a sample from the distribution *P*. In addition we are given *k* layers of environmental data for the region described by features *f*_*j*_(*X*) for *j* = 1, …, *k*. For example, one such function could be the average elevation in each geographic division.

Jaynes’ maximum entropy model attempts to reconstruct *P*. Let $\hat{P}=\left\{{\hat{p}}_{1},{\hat{p}}_{2},...,{\hat{p}}_{n}\right\}$ be the reconstructed density. Let $\hat{E}\left(f_{j}\left(X\right)\right)=\left(\sum_{i=1}^{n}o_{i}f_{j}\left(x_{i}\right)\right)/\left(\sum_{i=1}^{n}o_{i}\right)$ be the empirical estimate of $E_{P}\left(f_{j}\left(X\right)\right)=\sum_{i=1}^{n}p_{i}f_{j}\left(x_{i}\right)$ given by the occurrence data *O*. Jaynes’ maximum entropy model attempts to reconstruct *P* through an optimization problem. The optimization uses Shannon’s measure of entropy as the objective (1), subject to the moment constraints (2). Constraints (3) and (4) ensure that the optimal solution for the optimization is a probability distribution [9]. A mathematical formulation of the maximum entropy problem is

$$\begin{array}{c} \max_{p_{i}}\;-\sum_{i=1}^{n}p_{i}\log p_{i} \end{array}$$ (1)

$$\begin{array}{c} \text{s}.\text{t}.\;\sum_{i=1}^{n}p_{i}f_{j}\left(x_{i}\right)=\hat{E}\left(f_{j}\left(X\right)\right)\;j=1,\ldots ,k \end{array}$$ (2)

$$\begin{array}{c} \sum_{i=1}^{n}p_{i}=1 \end{array}$$ (3)

$$\begin{array}{c} p_{i}\geq 0\;i=1,\ldots ,n \end{array}$$ (4)

### Bootstrap method

Table 1 describes the bootstrap method for estimating the uncertainty of the estimate resulting from maximum entropy. The core of this bootstrap procedure is thinking of the distribution *P* as parameterized by the values it assigns to each geographic division. The procedure starts by estimating the parameters once, yielding a probability distribution. Then, it samples the data from that estimated distribution to construct several new estimates.

**Table 1:** Bootstrap method.

Algorithm	Bootstrapping
1	**function** Bootstrapping (*N*)
2	$\hat{P}=M\left(O\right)$
3	**For** *i* = 1: *N* **do**
4	${\hat{O}}_{n}=B\left(\hat{P},m\right)$
5	$P^{\prime }=M\left({\hat{O}}_{n}\right)$
6	Record *P*′
7	Return *SD*(*P*′, *N*)
*N*	Repeat the procedure *N* times
*O* = {*o*_1_, *o*_2_, …, *o*_*n*_}	Original occurrence data
*M*(*O*_*n*_)	Fit a maximum entropy model given a set of species occurrence data *O*_*n*_ = {*o*_1_, *o*_2_, …, *o*_*n*_} and return probability density estimation $\hat{p}$
$\hat{P}$ and *P*′	A reconstructed density over the geographic region
$B(\hat{p},m)$	Sample *m* occurrence data following probability density $\hat{p}$, where $m=\sum_{i=1}^{n}o_{i}$
${\hat{O}}_{n}=\left\{{\hat{o}}_{1}^{n},{\hat{o}}_{2}^{n},...,{\hat{o}}_{n}^{n}\right\}$	The *n*^*th*^ new sampled occurrence data with $\sum_{i=1}^{n}{\hat{o}}_{i}^{n}=\sum_{i=1}^{n}o_{i}$
*SD*(*P*′, *N*)	Calculate standard deviation of the set of *P*′*s*

### Analytic deduction of uncertainty

In this section, we demonstrate the basic idea of the analytical method for quantifying uncertainty in maximum entropy. The data *O* = {*o*_1_, *o*_2_, …*o*_*n*_} follow a multinomial distribution with unknown parameters *P*. A maximum likelihood estimator for *P* follows a certain multivariate normal distribution as the number of samples grow large. The maximum entropy model can be viewed as a function mapping this estimator to $R^{n}$. The input is the empirical expectations, $\hat{E}\left(f_{j}\left(X\right)\right)$, derived from the observation data, *O* = {*o*_1_, *o*_2_, …*o*_*n*_}. The output is the estimate of the probability distribution over geographic regions, *P* = {*p*_1_, *p*_2_, …, *p*_*n*_}. The analytical method of quantifying uncertainty describes how the output, *P*, changes as the input, *O*, changes. This is essentially a quantification of the way the optimization mapping warps the data input space, to the output space. We show the detailed deduction of the analytic method for uncertainty in the S1 Appendix.

For brevity, let $a_{j}=\hat{E}\left(f_{j}\left(X\right)\right)$ and the vector of *a*_*j*_ can be expressed as *A* = (*a*_1_, *a*_2_, …*a*_*k*_)^*T*^. Let *g*(*A*) denote the maximum entropy optimization, model (1),(2),(3),(4), as a function from $R^{k}$ to $R^{n}$. In other words, the function takes as input the vector *A* with *j*^*th*^ entry specified by *a*_*j*_, specifying right hand sides of the equality constraints $\hat{E}\left(f_{j}\left(X\right)\right)$, and outputs a probability estimate across the geographic region *P*. We would like to understand the uncertainty in the output *g*(*A*) as a function of the uncertainty of the input *A*. This can be done following steps similar to those in the delta method [19, p.75].

To understand the uncertainty in the output *g*(*A*), we begin by writing a first order Taylor expansion of *g* around *E*(*A*)

$$\begin{array}{ll} g\left(A\right) & \approx g\left(E\left(A\right)\right)+\nabla g\left(E\left(A\right)\right)\cdot \left[A-E\left(A\right)\right]\\ & \approx g\left(\text{F}\cdot \hat{P}\right)+\nabla g\left(\text{F}\cdot \hat{P}\right)\cdot \left[A-E\left(A\right)\right], \end{array}$$ (5)

where **F** is *k* × *n* matrix of *k* features with entry (*i*, *j*) specified by *f*_*i*_(*x*_*j*_) and ∇*g*(⋅) is an *n* × *k* matrix of partial derivatives, with entry (*i*, *j*) specified by $\frac{\partial p_{i}}{\partial a_{j}}$. If we can compute an expression for these partial derivatives, then everything on the right hand side above is constant, except [*A* − *E*(*A*)] whose distribution we know because we know the distribution of *A*. *g*(*A*) is an affine transformation of [*A* − *E*(*A*)], and can be approximated as

$$\begin{array}{c} g\left(A\right)∼\text{Normal}(g\left(F\cdot \hat{P}\right),\nabla g\cdot \frac{F\cdot \Sigma \cdot F^{T}}{m}\cdot {\left(\nabla g\right)}^{T}), \end{array}$$ (6)

where **Σ** is proportional to the covariance matrix of $\hat{P}$ with entry (*i*, *j*) specified by $-{\hat{p}}_{i}{\hat{p}}_{j}$ for *i* ≠ *j*, and entry (*i*, *i*) specified by ${\hat{p}}_{i}\left(1-{\hat{p}}_{i}\right)$.

We express the $\frac{\partial p_{i}}{\partial a_{j}}$ as (Detailed deduction shown in S1 Appendix)

$$\begin{array}{c} \frac{\partial p_{i}}{\partial a_{j}}=\sum_{r=1}^{k}p_{i}\left(a_{r}-f_{r}\left(x_{i}\right)\right){\left({\left(-\Psi \right)}^{-1}\right)}_{rj}, \end{array}$$ (7)

where **Ψ**_*rj*_ = *cov*_*P*_(*f*_*r*_, *f*_*j*_) is the covariance matrix of features with respect to the maximum entropy model results, and *f*_*j*_ denotes the *j*^*th*^ feature in constraint (2). We denote the inverse covariance matrix as **Ψ**^−1^ and refer to its (*r*, *j*)th entry as (**Ψ**^−1^)_*rj*_.

To summarize, one can compute analytical estimates of the uncertainty as follows:

1. Gather data for *f*_*r*_ (⋅) and the right-hand sides of constraints (2), *a*_*r*_.
2. Solve the maximum entropy model to get a vector of *P* of probabilities *p*_*i*_.
3. Compute the matrix −*cov*_*P*_(*f*_*r*_, *f*_*j*_), using the features and the vector *P*.
4. Compute the derivates $\frac{\partial p_{i}}{\partial a_{j}}$ using (7), giving the matrix ∇*g*.
5. The covariance of the output *P* can then be estimated as $\nabla g\cdot \frac{F\cdot \Sigma \cdot F^{T}}{m}\cdot {\left(\nabla g\right)}^{T}$, following Eq (5).

## Results

We demonstrate the applications of the analytical expression of the uncertainty through two examples, Dengue virus and Aedes Aegypti mosquito, and compare the analytical results with the uncertainty calculated using the bootstrap method and Poission PPM approach. The analytic method results aligned well with bootstrap method results, but Poisson PPM approach gave much larger standard deviations. We only show the results and comparison of analytic and bootstrap below but include results and comparison of Poisson PPM in S1 Figs. The resolution of the Dengue virus example is at county level while the resolution of the Aedes Aegypti mosquito is at 1 *km*^2^ area level through Texas.

### Dengue importation probabilities

Dengue virus is often imported into Texas from endemic counties. We aim to estimate the probability that the next importation case will happen in each county of Texas. Historical case import data, *O* = {*o*_1_, *o*_2_, …*o*_*n*_} with *n* equal to 254 counties in Texas, present empirical samples from this distribution. Each *o*_*i*_ counts the number of imports in county *i*. We are also given features *f*_*j*_(*X*) ∈ *R*^1×254^ for *j* = 1, …, 10 that represent socio-economic, demographic, and environmental features selected for all 254 counties across the Texas counties. This completely defines the inputs necessary for a maximum entropy model.

**Fig 1:**
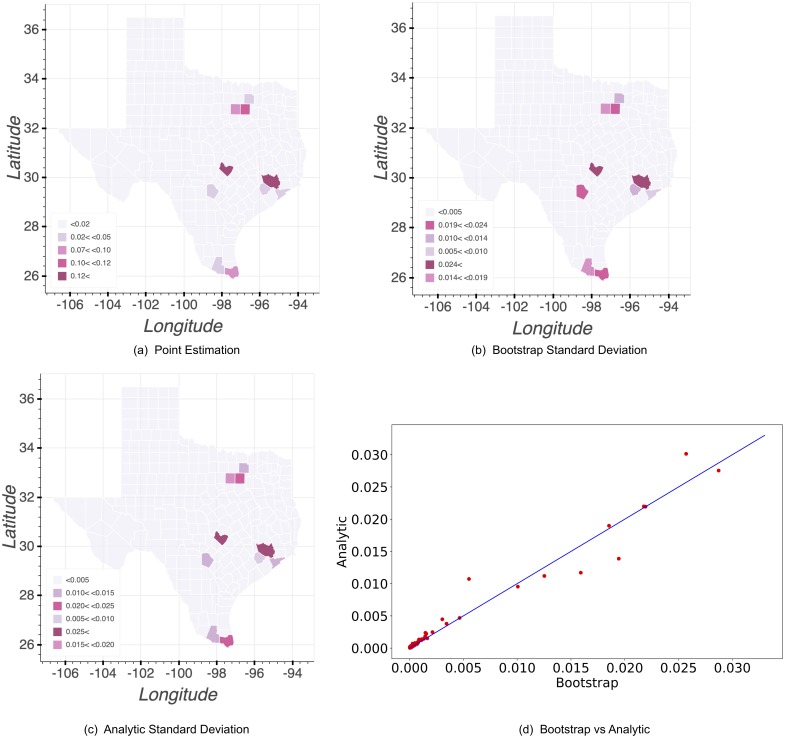
Standard deviation comparison for Dengue importation probability. (a) Figure shows the point estimates for the import probability ${\hat{p}}_{i}$. (b) Figure visually plots the bootstrap standard deviation estimates for *p*_*i*_ across Texas counties. (c) Figure visually plots the analytic standard deviation estimates for *p*_*i*_ across Texas counties. (d) Figure plots the standard deviations of bootstrap vs. analytic and shows a strong equivalence between the two. Each red dot represent the estimations for one county.

Specifically, we use ten years, 2002 to 2012, of Dengue importation data into Texas received from the Texas Department of State Health Services. The features *f*_*j*_(*X*) represent features listed in Table 2. The ten final features were selected through a series feature selection procedures, including representative variable selections and most predictive variable selections, which demonstrated in [20]. We estimate the standard deviation using the bootstrap method, Poission PPM approach and the analytic method. The results are presented in Fig 1.

**Table 2:** Ten features included in maximum entropy model. The data for these features is derived at a county level from the 2009-2013 American Community Survey 5-year estimates [21] and WorldClim Database [22].

*Features*
Population of Educational Attainment with Bachelor’s degree
Minimum Temperature of Coldest Month
Percentage of Using Public Transportation to Work
Population of Educational Attainment in some college(no degree)
Population of Walked to Work
Population of Commuting to Work with Other Means
Population of Educational Attainment less than 9th grade
Percentage with Graduate or professional degree
Percentage of Walked to Work
Average Artificial Surface (Percentage)

Fig 1a shows the point estimates for the import probability estimated from maximum entropy model and Fig 1b represents standard deviation estimates from the bootstrap method and analytic method of maximum entropy model, respectively. Many Texas counties have never had imported Dengue cases over the past ten years, and their estimates are close to zero. We map the standard deviation of the estimates *p*_*i*_ of each county in Fig 1b and 1c with a darker color indicating a higher standard deviation level. For bootstrap method, we did 2000 bootstrap runs and took 22403.34 seconds in total. The running time of the analytic method, using optimized matrix operations as described in the S1 Appendix, is dramatically faster than the bootstrap method and takes 0.0016 seconds in total.

Fig 1d shows the standard deviation resulting from the bootstrap against the standard deviation resulting from the analytic method. Each red dot represent a county. It also depicts a regression line between the two results—*s*_*a*_ = 0.98*s*_*b*_ with *R*^2^ = 0.972, where *s*_*a*_ and *s*_*b*_ stand for the standard deviation estimates from the analytic and the bootstrap methods, respectively. Regression results show a linear relationship between the standard deviation calculated from analytic expression and bootstrap method with parameter approximate 1. Both bootstrap method and analytic method generally indicate larger standard deviation for counties with larger point estimates.

### Aedes aegypti habitat

The Aedes aegypti mosquito is the primary transmission vector of dengue, chikungunya, and zika viruses. We aim to estimate the relative probability distribution of Aedes aegypti in Texas. Historical presence data *O* = {*o*_1_, *o*_2_, …*o*_*n*_}, with *n* equal to the number of 1km grid squares in Texas, present empirical samples from this distribution. Each *o*_*i*_ is either 0, if there is no presence data for this square, or 1 if there is presence data. The features *f*_*j*_(*X*) represent environmental data for each 1 *km*^2^ area across the Texas.

Specifically, we use 121 locations, within Texas, of Aedes aegypti presence data found from previous studies [23–30], DSHS. The environmental features *f*_*j*_(*X*), found from WorldClim Database [22], are listed in Table 3.

**Table 3:** Seven features, found from WorldClim Database [22], included in maximum entropy model.

*Features*
artificial surfaces
population count
temperature seasonality
elevation
precipitation seasonality
minimum temperature of coldest month
mean diurnal range

**Fig 2:**
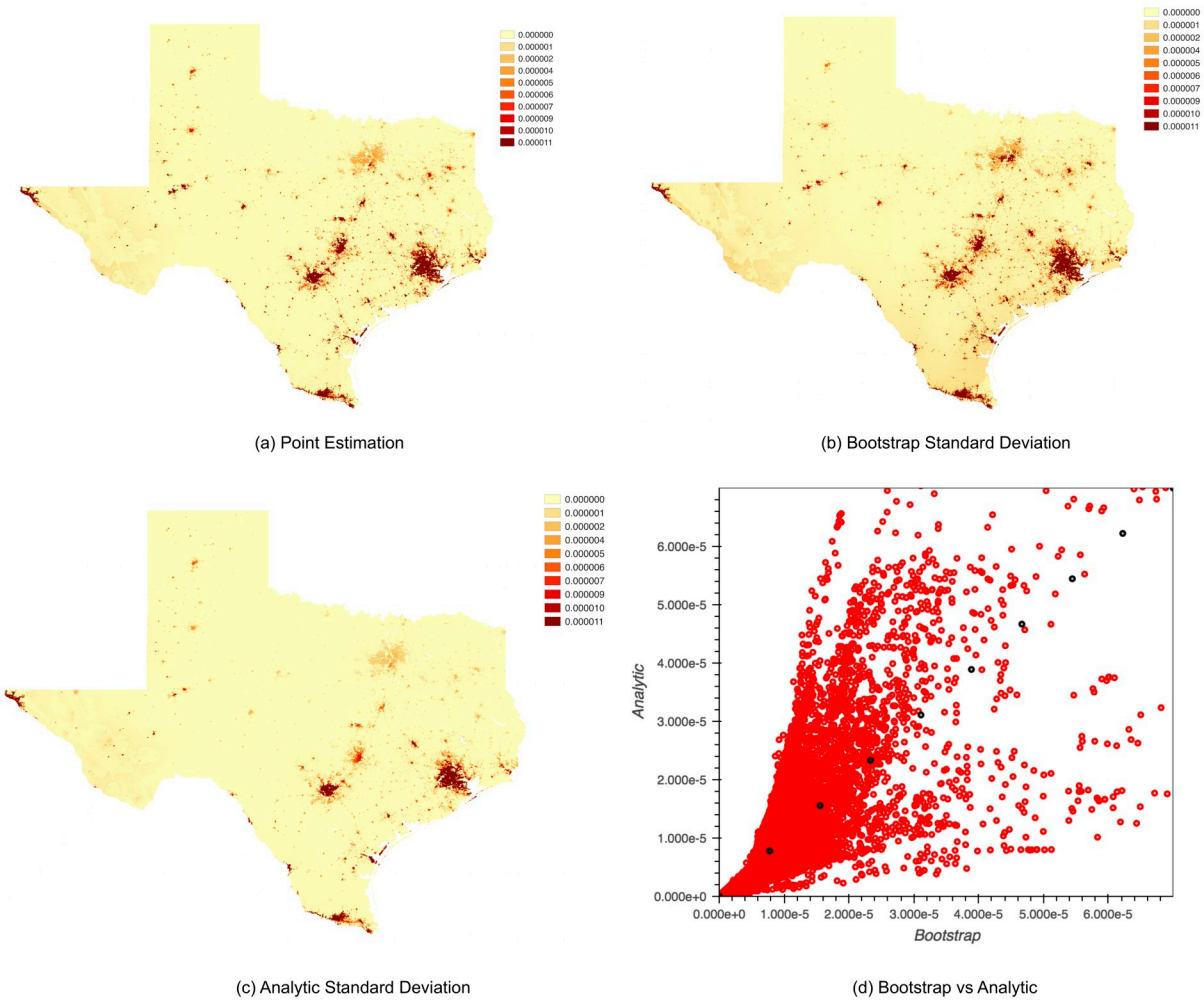
Standard deviation comparison for Aedes aegypti. (a) Figure presents the point estimates *p*_*i*_. (b) Figure shows standard deviation calculated using bootstrap method. (c) Figure shows standard deviation calculated using analytic method. (d) Figure shows the standard deviation comparison between analytic method and bootstrap method.

We aim to analyze the standard deviation of the estimates ${\hat{p}}_{i}$ for each 1km square. We estimate this standard deviation using both the bootstrap method and the analytic method. The results are presented in Fig 2.

We present the point estimates of the distribution of the Aedes aegypti mosquito in Fig 2a. Aedes aegypti primarily feeds on humans and is found in urban areas, which results in higher probability estimates in those areas. The areas of concentration of Aedes aegypti in Texas tend to be population centers like Houston, Dallas, San Antonio, Austin, El Paso, and McAllen.

Fig 2b and 2c plots the standard deviation of the estimates *p*_*i*_ of each grid using the bootstrap method and the analytic method. This can give a practitioner a good sense of the standard deviation in the estimates. In applying the analytic method, one could use as input the empirical distribution or a Laplace smoothed estimator [31] to smooth the empirical probability to be non-zero. The analytic method gives slightly higher uncertainty estimates than bootstrap as shown in Fig 2c. Each red dot represent the standard deviation estimates for each grid using the bootstrap and the analytic method respectively. The black dot shows the diagonal line when two methods aligned well. When we applied a Laplace smoothing of 0.0001, we have the relationship *s*_*a*_ = 1.0744*s*_*b*_ with *R*^2^ = 0.802, where *s*_*a*_ and *s*_*b*_ stand for the standard deviation estimates from the analytic and the bootstrap methods, respectively. We map the standard deviation of the estimates *p*_*i*_ of each 1 *km*^2^ using the analytic method and Laplace smoothing of 0.0001 in Fig 2b. The bootstrap result and analytic result can be visually compared through Fig 2c.

We did 2000 bootstrap runs and took 30400 seconds in total. The running time for the analytic method is 6516 seconds, which is much faster than bootstrap method. As we calculated the relative probability for Aedes aegypti for a 1*km*^2^ square grid, we have 933,680 grid cells in total. Computing the covariance of the output would require matrix multiplications for matrices of size 933680 × 933680, which can cause out-of-memory errors. We introduce a faster method of calculating the variance of each square grid in S1 Appendix.

## Discussion

The maximum entropy model can give a point estimation of the unknown species distribution within predefined grids using presence-only data with possible influential features like environmental factors, demographic factors, social economic factors, etc. However, uncertainties come from both the model and the sample data. Some possible sources of this uncertainty are:

- The true expectation of all features *f*_*j*_(*X*) are unknown and estimated using the presence-only data.
- Species distribution data are not collected at random, but based on prior knowledge of the biologists. For example, all samples may be observed within pre-selected locations.
- Having only a few presence points relative to the size of the grid can lead to unstable models.
- The features *f*_*j*_(*X*) used within the model may be inaccurate or vary dramatically over time. So, it is unclear whether the presence only data collected is appropriate for use with the given features.

In the maximum entropy model, the output probabilities are dependent on the features *f*_*j*_(*X*). A flat *f*_*j*_(*X*) can only produce flat output probabilities. One may want to know how will the output probabilities change when the feature values change? Uncertainty quantification may help identify the features that most reduce uncertainty in a maximum entropy model.

The bootstrap method is a well accepted method of quantifying uncertainty. However, the running time of the bootstrap can be very long. In the dengue example, bootstrap method took more than 22000 seconds to generate a comparable uncertainty estimate of analytic method while the analytic method just took 0.0016 seconds in total. The analytic method uses more memory compared to the bootstrap method. In the Aedes aegypti example, the analytic method took only 20 percent time of running bootstrap method. However, code optimization, and element-wise matrix multiplications can significantly increase the speed of the analytic method compared to the bootstrap method. A method for increasing programming speed are shown in S1 Appendix. Furthermore, the analytic method is able to approximate covariances in the output—whereas this can be quite difficult for the bootstrap method if we only use a small number of samples.

The Poisson PPM approach proved to be equivalent to MAXENT providing an alternative approach of estimating the uncertainty. However, the hidden independence assumption of species appearance locations can affect the performance of the model which gives much larger estimated uncertainty when assumptions violated.

## Supporting information

S1 Appendix Analytic expression of uncertainty, and comparison between analytic method and poisson PPM can be found in S1 Appendix. (PDF)

S1 Figs Analytic and poisson PPM comparison. (a) Figure plots the relationship between point estimates of Dengue importation probability vs. variance calculated through analytic method. Non-linear relationship indicates the improper use of Poisson PPM for Dengue importation cases. (b) Figure plots the standard deviations of Poisson PPM vs. analytic for Dengue importation case study and indicates that Poisson PPM provides much larger standard deviation for Dengue imports application. (c) Figure plots the relationship between point estimates of Aedes Aegypti existence probability vs. variance calculated through analytic method. (d) Figure shows the standard deviation comparison between analytic method and Poisson PPM of Aedes Aegypti existence probability.
(TIF)

S1 Data File Dengue importation and Aedes aegypti existence case study data. All the data used for Dengue importation and Aedes Aegypti existence case study are all included in data file.
(ZIP)
