# Correction: Uncertainty analysis of species distribution models

**DOI:** 10.1371/journal.pone.0226663

**Published:** 2019-12-12

**Authors:** Xi Chen, Lauren A. Castro, Nedialko B. Dimitrov, Lauren Ancel Meyers

Dr. Lauren A. Castro should be included in the author byline. They should be listed as the second author, and their affiliation is 2 Department of Integrative Biology, The University of Texas at Austin, Austin, TX, USA. The contributions of this author are as follows: Data curation. The correct citation is: Chen X, Castro LA, Dimitrov NB, Meyers LA (2019) Uncertainty analysis of species distribution models. PLoS ONE 14(5): e0214190. https://doi.org/10.1371/journal.pone.0214190
