# Supplementary material for: Uncertainty analysis of species distribution models
Source: PLoS One. 2019 May 23;14(5):e0214190. doi: 10.1371/journal.pone.0214190 (PMC6533036; doi:10.1371/journal.pone.0214190)
Supplement: S1 Appendix — (PDF) [file pone.0214190.s001.pdf]

S1 Appendix

Analytic Expression of Uncertainty

Consider a region with geographic divisions given by $X = \{x_1, x_2, ..., x_n\}$. Suppose some species lives in the region, and the fraction of the species that lives in division i is p_i. A basic goal in SDM is to reconstruct the geographic distribution $P = \{p_1, p_2, ..., p_n\}$. To do this, we have some species occurrence data $O = \{o_1, o_2, ..., o_n\}$, where each o_i specifies the number of times the species has occurred in division i. The occurrence data can be viewed as a sample from the distribution P. In addition we are given k layers of environmental data for the region described by features $f_j(X)$ for $j = 1, \ldots, k$. For example, one such function could be the average elevation in each geographic division.

The mathematical formulation of the maximum entropy problem is

$$\max_{p_i} \quad -\sum_{i=1}^{n} p_i \log p_i \tag{1a}$$

$$\text{s.t.} \quad \sum_{i=1}^{n} p_i f_j(x_i) = \hat{E}(f_j(X)) \quad j = 1, \ldots, k \tag{1b}$$

$$\sum_{i=1}^{n} p_i = 1 \tag{1c}$$

$$p_i \geq 0 \qquad i = 1, \ldots, n \tag{1d}$$

The counts $O = \{o_1, o_2, ...o_n\}$ follow a multinomial distribution, whose true parameter $P = \{p_1, p_2, ..., p_n\}$ is unknown. Let $m = \sum_{i=1}^{n} o_i$ be the total number of observations. The maximum likelihood estimator of $P = \{p_1, p_2, ..., p_n\}$, is

$$\tilde{P} = \begin{pmatrix} \tilde{p}_1 \\ \tilde{p}_2 \\ \vdots \\ \tilde{p}_n \end{pmatrix} = \frac{1}{m} \begin{pmatrix} o_1 \\ o_2 \\ \vdots \\ o_n \end{pmatrix} = \frac{O}{m}$$

and it follows a normal distribution [1],

$$\tilde{P} \sim \text{Normal}(P, \frac{\mathbf{\Sigma}}{m}),$$

where

$$\mathbf{\Sigma} = \begin{pmatrix} p_1(1-p_1) & -p_1p_2 & \cdots & -p_1p_n \\ -p_1p_2 & p_2(1-p_2) & \cdots & -p_2p_n \\ \vdots & \vdots & \ddots & \vdots \\ -p_1p_n & -p_2p_n & \cdots & p_n(1-p_n) \end{pmatrix}.$$

From the analysis in Kapur et al. [2], one can show that the maximum likelihood estimates of P from maximum entropy model (1) are achieved when

$$\hat{E}(f_j(X)) = \frac{\sum_{i=1}^{n} f_j(x_i) o_i}{\sum_{i=1}^{n} o_i} \tag{2}$$

For brevity, let $a_j = \hat{E}(f_j(X))$. Based on equation (2), the vector of a_j can be expressed as

$$A = \begin{pmatrix} a_1 \\ a_2 \\ \vdots \\ a_k \end{pmatrix} = \frac{1}{m} \begin{pmatrix} f_1(x_1) & f_1(x_2) & \cdots & f_1(x_n) \\ f_2(x_1) & f_2(x_2) & \cdots & f_2(x_n) \\ \vdots & \vdots & \ddots & \vdots \\ f_k(x_1) & f_k(x_2) & \cdots & f_k(x_n) \end{pmatrix} \begin{pmatrix} o_1 \\ o_2 \\ \vdots \\ o_n \end{pmatrix} \tag{3}$$

Let

$$\mathbf{F} = \begin{pmatrix} f_1(x_1) & f_1(x_2) & \cdots & f_1(x_n) \\ f_2(x_1) & f_2(x_2) & \cdots & f_2(x_n) \\ \vdots & \vdots & \ddots & \vdots \\ f_k(x_1) & f_k(x_2) & \cdots & f_k(x_n) \end{pmatrix},$$

then

$$A = \frac{1}{m} \cdot \mathbf{F} \cdot O = \mathbf{F} \cdot \tilde{P}$$

Because $\mathbf{F}$ is constant, A is an affine transformation of $\tilde{P}$. Using the distribution of $\tilde{P}$, we can write the distribution of A [3]

$$A \sim \text{Normal}(\mathbf{F} \cdot P, \frac{\mathbf{F} \cdot \mathbf{\Sigma} \cdot \mathbf{F}^T}{m}). \tag{4}$$

Let $g(A)$ denote the maximum entropy optimization, model (1), as a function from $\mathcal{R}^k$ to $\mathcal{R}^n$. In other words, the function takes as input the vector A, specifying right hand sides of the equality constraints $\hat{E}(f_j(X))$, and outputs a probability estimate across the geographic region P. We would like to understand the uncertainty in the output $g(A)$ as a function of the uncertainty of the input A. This can be done following steps similar to those in the delta method [4, p.75].

To understand the uncertainty in the output $g(A)$, we begin by writing a first order Taylor expansion of g around $E(A)$

$$\begin{aligned} g(A) &\approx g(E(A)) + \nabla g(E(A)) \cdot [A - E(A)] \\ &\approx g(\mathbf{F} \cdot P) + \nabla g(\mathbf{F} \cdot P) \cdot [A - E(A)], \end{aligned} \tag{5}$$

where $\nabla g(\cdot)$ is an $n \times k$ matrix of partial derivatives, with entry (i, j) specified by $\frac{\partial p_i}{\partial a_j}$. If we can compute an expression for these partial derivatives, then everything on the right hand side above is constant, except $[A - E(A)]$ whose distribution we know because we know the distribution of A. $g(A)$ is an affine transformation of $[A - E(A)]$, and can be approximated as

$$g(A) \sim \text{Normal}(g(\mathbf{F} \cdot P), \nabla g \cdot \frac{\mathbf{F} \cdot \mathbf{\Sigma} \cdot \mathbf{F}^T}{m} \cdot (\nabla g)^T). \tag{6}$$

To complete the analysis of the output uncertainty, we continue by deriving an expression for $\frac{\partial p_i}{\partial a_j}$. We introduce some additional notation, following Kapur et al. [2]. Let λ be the Lagrange multiplier for constraint (1c), and μ_j be the multiplier for constraint (1b) for $j = 1, \ldots, k$. It can be shown [2] that the optimal p_i have the expression

$$p_i = e^{-\sum_{j=1}^{k} \mu_j f_j(x_i) - \lambda - 1} \qquad \forall i = 1, 2, ...n. \tag{7}$$

Using the constraint (1c), we have

$$\sum_{t=1}^{n} e^{-\lambda-1-\sum_{j=1}^{k} f_j(x_t)\mu_j} = 1$$

$$e^{\lambda+1} = \sum_{t=1}^{n} e^{-\sum_{j=1}^{k} f_j(x_t)\mu_j}. \tag{8}$$

We can now substitute the expression for $e^{\lambda+1}$ back into (7) to derive

$$p_i = \frac{e^{-\sum_{j=1}^{k} f_j(x_i)\mu_j}}{\sum_{t=1}^{n} e^{-\sum_{j=1}^{k} f_j(x_t)\mu_j}} \tag{9}$$

We now have an expression of the p_i in terms of the dual multipliers μ_j. But, we would like to compute $\frac{\partial p_i}{\partial a_j}$, which we can do by first computing partial derivatives with respect to the μ_j and using the expression

$$\frac{\partial p_i}{\partial a_j} = \sum_{r=1}^{k} \frac{\partial p_i}{\partial \mu_r} \frac{\partial \mu_r}{\partial a_j}.$$

What remains to be computed is $\frac{\partial p_i}{\partial \mu_r}$ and $\frac{\partial \mu_r}{\partial a_j}$. From (9), we have

$$\begin{aligned}
\frac{\partial p_i(\mu_1, \mu_2 ... \mu_m)}{\partial \mu_r} &= -f_r(x_i) e^{-\sum_{j=1}^{k} f_j(x_i)\mu_j} (\sum_{t=1}^{n} e^{-\sum_{j=1}^{k} f_j(x_t)\mu_j})^{-1} \\
&\quad - (\sum_{t=1}^{n} e^{-\sum_{j=1}^{k} f_j(x_t)\mu_j})^{-2} (\sum_{t=1}^{n} (-1) f_r(x_t) e^{-\sum_{j=1}^{k} f_j(x_t)\mu_j}) e^{-\sum_{j=1}^{k} f_j(x_i)\mu_j} \\
&= -f_r(x_i) p_i - p_i (\sum_{t=1}^{n} (-1) f_r(x_t) p_t) \\
&= -f_r(x_i) p_i + p_i a_r \\
&= p_i (a_r - f_r(x_i)),
\end{aligned}$$

where we derived the first equality from the chain rule, the second equality by substituting using expression (9), and the third equality by using the fact that $a_r = \sum_{t=1}^{n} f_r(x_t) p_t$ because p_i's are feasible and optimal in the maximum entropy optimization.

Then, we want to get the value of $\frac{\partial \mu_r}{\partial a_j}$. It is hard to get the expression of μ_r in terms of a_j, however, we can derive $\frac{\partial a_j}{\partial \mu_r}$ and use the Inverse Function Theorem [5] to calculate $\frac{\partial \mu_r}{\partial a_j}$. To get the expression of a_j in terms of μ_r, we substitute the expression of the optimal p_i (7) into constraint (1b), and then plug-in the expression of λ in terms of μ_r (8). Then, we express a_j as

$$a_j = \frac{\sum_{t=1}^{n} f_j(x_t) e^{-\sum_{j=1}^{k} f_j(x_t)\mu_j}}{\sum_{t=1}^{n} e^{-\sum_{j=1}^{k} f_j(x_t)\mu_j}}. \tag{10}$$

From 10, we have

$$\frac{\partial a_j}{\partial \mu_r}$$
$$= \sum_{i=1}^{n}(-f_r(x_i))f_j(x_i)e^{-\sum_{j=1}^{m} f_j(x_i)\mu_j}(\sum_{i=1}^{n} e^{-\sum_{j=1}^{k} f_j(x_i)\mu_j})^{-1}$$
$$-(\sum_{i=1}^{n} e^{-\sum_{j=1}^{m} f_j(x_i)\mu_j})^{-2}(\sum_{i=1}^{n} f_j(x_i)e^{-\sum_{j=1}^{k} f_j(x_i)\mu_j})(\sum_{i=1}^{n} -f_r(x_i)e^{-\sum_{j=1}^{k} f_j(x_i)\mu_j})$$
$$= -\sum_{i=1}^{n} f_r(x_i)f_j(x_i)p_i + (\sum_{i=1}^{n} f_j(x_i)p_i)(\sum_{i=1}^{n} f_r(x_i)p_i)$$
$$= -cov_P(f_r, f_j),$$

where we derived the first equality from the chain rule, the second equality by substituting using expression (9). The final equality comes from the definition of covariance, where we take the covariance of feature f_r and f_j with respect to the maximum entropy model results p_i.

If the determinant of the covariance is non-zero, following the Inverse Function Theorem [5], the inverse is differentiable. We denote the covariance matrix of features with respect to the maximum entropy model results as $\mathbf{\Psi}$, where $\mathbf{\Psi}_{rj} = cov_P(f_r, f_j)$. We denote the inverse covariance matrix as $\mathbf{\Psi}^{-1}$ and refer to its (r, j)th entry as $(\mathbf{\Psi}^{-1})_{rj}$. By the inverse function theorem, $\frac{\partial \mu_r}{\partial a_j}$ is equal to $(\mathbf{\Psi}^{-1})_{rj}$. Finally, we can express the $\frac{\partial p_i}{\partial a_j}$ as

$$\frac{\partial p_i}{\partial a_j} = \sum_{r=1}^{k} p_i(a_r - f_r(x_i))(-\mathbf{\Psi}^{-1})_{rj}. \tag{11}$$

Increasing programming speed

In the calculation of the relative probability for Aedes aegypti for a $1km^2$ square grid, we have 933,680 grid cells in total. The problem of computing the covariance of the output mainly comes from $\mathbf{\Sigma}$ where

$$\mathbf{\Sigma} = \begin{pmatrix} \hat{p}_1(1-\hat{p}_1) & -\hat{p}_1\hat{p}_2 & \cdots & -\hat{p}_1\hat{p}_n \\ -\hat{p}_1\hat{p}_2 & \hat{p}_2(1-\hat{p}_2) & \cdots & -\hat{p}_2\hat{p}_n \\ \vdots & \vdots & \ddots & \vdots \\ -\hat{p}_1\hat{p}_n & -\hat{p}_2\hat{p}_n & \cdots & \hat{p}_n(1-\hat{p}_n) \end{pmatrix}.$$

The matrix is of size 933680×933680, which can cause out of memory errors.

To figure out a way of speeding the calculation, we first split $\mathbf{\Sigma}$ into two parts, where

$$\mathbf{\Sigma} = \begin{pmatrix} \hat{p}_1(1-\hat{p}_1) & -\hat{p}_1\hat{p}_2 & \cdots & -\hat{p}_1\hat{p}_n \\ -\hat{p}_1\hat{p}_2 & \hat{p}_2(1-\hat{p}_2) & \cdots & -\hat{p}_2\hat{p}_n \\ \vdots & \vdots & \ddots & \vdots \\ -\hat{p}_1\hat{p}_n & -\hat{p}_2\hat{p}_n & \cdots & \hat{p}_n(1-\hat{p}_n) \end{pmatrix}$$
$$= \begin{pmatrix} -\hat{p}_1^2 & -\hat{p}_1\hat{p}_2 & \cdots & -\hat{p}_1\hat{p}_n \\ -\hat{p}_1\hat{p}_2 & -\hat{p}_2^2 & \cdots & -\hat{p}_2\hat{p}_n \\ \vdots & \vdots & \ddots & \vdots \\ -\hat{p}_1\hat{p}_n & -\hat{p}_2\hat{p}_n & \cdots & -\hat{p}_n^2 \end{pmatrix} + \begin{pmatrix} \hat{p}_1 & 0 & \cdots & 0 \\ 0 & \hat{p}_2 & \cdots & 0 \\ \vdots & \vdots & \ddots & \vdots \\ 0 & 0 & \cdots & \hat{p}_n \end{pmatrix}.$$

Define

$$\mathbf{\Sigma}_1 = \begin{pmatrix} -\hat{p}_1^2 & -\hat{p}_1\hat{p}_2 & \cdots & -\hat{p}_1\hat{p}_n \\ -\hat{p}_1\hat{p}_2 & -\hat{p}_2^2 & \cdots & -\hat{p}_2\hat{p}_n \\ \vdots & \vdots & \ddots & \vdots \\ -\hat{p}_1\hat{p}_n & -\hat{p}_2\hat{p}_n & \cdots & -\hat{p}_n^2 \end{pmatrix},$$

and

$$\mathbf{\Sigma}_2 = \begin{pmatrix} \hat{p}_1 & 0 & \cdots & 0 \\ 0 & \hat{p}_2 & \cdots & 0 \\ \vdots & \vdots & \ddots & \vdots \\ 0 & 0 & \cdots & \hat{p}_n \end{pmatrix}.$$

The covariance of the output P can be estimated as

$$\nabla g \cdot \frac{\mathbf{F} \cdot \mathbf{\Sigma} \cdot \mathbf{F}^T}{m} \cdot (\nabla g)^T = \nabla g \cdot \frac{\mathbf{F} \cdot (\mathbf{\Sigma}_1 + \mathbf{\Sigma}_2) \cdot \mathbf{F}^T}{m} \cdot (\nabla g)^T.$$

First, to calculate $\nabla g \cdot \frac{\mathbf{F} \cdot \mathbf{\Sigma}_1 \cdot \mathbf{F}^T}{m} \cdot (\nabla g)^T$, we define

$$P_1 = \begin{pmatrix} \hat{p}_1 & 0 & \cdots & 0 \\ \hat{p}_2 & 0 & \cdots & 0 \\ \vdots & \vdots & \ddots & \vdots \\ \hat{p}_n & 0 & \cdots & 0 \end{pmatrix},$$

then we have

$$\nabla g \cdot \frac{\mathbf{F} \cdot \mathbf{\Sigma}_1 \cdot \mathbf{F}^T}{m} \cdot (\nabla g)^T = \nabla g \cdot \frac{\mathbf{F} \cdot P_1 \cdot P_1^T \cdot \mathbf{F}^T}{m} \cdot (\nabla g)^T.$$

The ith diagonal element of $\nabla g \cdot \mathbf{F} \cdot \Sigma_1 \cdot \mathbf{F}^T \cdot (\nabla g)^T$, denoted as d_i^1, can be calculated as

$$\begin{aligned} d_i^1 &= \left(\frac{\partial p_i}{\partial a_1}(f_1(x_1)\hat{p_1} + \cdots + f_1(x_n)\hat{p_n}) + \cdots + \frac{\partial p_i}{\partial a_k}(f_k(x_1)\hat{p_1} + \cdots + f_k(x_n)\hat{p_n}) \right)^2 \\ &= \left(\sum_{\ell=1}^{k} \frac{\partial p_i}{\partial a_\ell} (\sum_{m=1}^{n} f_\ell(x_m)\hat{p_m}) \right)^2 \end{aligned} \quad (12)$$

To calculate $\nabla g \cdot \frac{\mathbf{F} \cdot \mathbf{\Sigma}_2 \cdot \mathbf{F}^T}{m} \cdot (\nabla g)^T$, we define

$$P_2 = \begin{pmatrix} \sqrt{\hat{p}_1} & 0 & \cdots & 0 \\ 0 & \sqrt{\hat{p}_2} & \cdots & 0 \\ \vdots & \vdots & \ddots & \vdots \\ 0 & 0 & \cdots & \sqrt{\hat{p}_n} \end{pmatrix},$$

then we have

$$\nabla g \cdot \frac{\mathbf{F} \cdot \mathbf{\Sigma}_2 \cdot \mathbf{F}^T}{m} \cdot (\nabla g)^T = \nabla g \cdot \frac{\mathbf{F} \cdot P_2 \cdot P_2^T \cdot \mathbf{F}^T}{m} \cdot (\nabla g)^T.$$

Fig 1. Analytic and Poisson PPM Comparison (a) Figure plots the relationship between point estimates of Dengue importation probability vs. variance calculated through analytic method. Non-linear relationship indicates the improper use of Poisson PPM for Dengue importation cases. (b) Figure plots the standard deviations of Poisson PPM vs. analytic for Dengue importation case study and indicates that Poisson PPM provides much larger standard deviation for Dengue imports application. (c) Figure plots the relationship between point estimates of Aedes Aegypti existence probability vs. variance calculated through analytic method. (d) Figure shows the standard deviation comparison between analytic method and Poisson PPM of Aedes Aegypti existence probability.

The ith diagonal element of $\nabla g \cdot \mathbf{F} \cdot \Sigma_2 \cdot \mathbf{F}^T \cdot (\nabla g)^T$, denoted as d_i^1, is calculated by

$$\begin{aligned} d_i^2 &= (\frac{\partial p_i}{\partial a_1} f_1(x_1)\sqrt{\hat{p_1}} + \cdots + \frac{\partial p_i}{\partial a_k} f_k(x_1)\sqrt{\hat{p_1}})^2 + \cdots + \\ &\qquad (\frac{\partial p_i}{\partial a_1} f_1(x_n)\sqrt{\hat{p_n}} + \cdots + \frac{\partial p_i}{\partial a_k} f_k(x_n)\sqrt{\hat{p_n}})^2 \\ &= \sum_{m=1}^{n} \left(\sum_{\ell=1}^{k} \frac{\partial p_i}{\partial a_\ell} f_\ell(x_m)\sqrt{\hat{p_m}} \right)^2 \end{aligned} \tag{13}$$

After we have calculated both d_i^1 and d_i^2 based on 12 and 13, we can calculate the variance of the p_i as $\frac{(d_i^1 + d_i^2)}{m}$.

Comparison between Analytic method and Poisson PPM

Poisson PPM was proved to be equivalent to maximum entropy model with hidden assumptions of independence data. We showed the plots of variance vs. point estimations and standard deviation comparison between Poisson PPM and analytic method for both Dengue importation probability and Aedes Aegypti suitability probability. Fig 1a shows the relationship between Dengue importation probability point estimates and estimated variance using analytic method, which indicating the possible improper use of Poisson PPM approach. Fig 1b shows a standard deviation comparison between the analytic and Poisson PPM method for Dengue importation probability. The regression line between two results is $s_a = 0.054 s_p$ with $R^2 = 0.805$, where s_a and s_p stand for the standard deviation estimates from the analytic and Poisson PPM methods, respectively. Poisson PPM gives a much larger standard deviation comparing to analytic and bootstrap method indicating the possible violate of the case location independence assumption.

Fig 1c shows the relationship between Aedes Aegypti suitability point estimates and estimated variance using analytic method. There is more linear relationship comparing to Dengue importation cases. Fig 1d shows the standard deviation comparison between analytic method and Poisson PPM approach for Aedes Aegypti suitability. Each red dot represent the standard deviation estimates for each grid using Poisson PPM and analytic method respectively. The blue dot shows the diagonal line when two methods aligned well. We have the relationship $s_a = 0.0917 s_p$ with $R^2 = 0.812$, where s_a and s_p stand for the standard deviation estimates from the analytic and Poisson PPM methods, respectively. Similar as Dengue case, Poisson PPM doesn't seem to functional well with a much larger standard deviation estimation comparing to analytic and bootstrap method.

References

1. Wasserman L. All of statistics: a concise course in statistical inference. Springer Science & Business Media; 2013.
2. Kapur JN, Kesavan HK. Entropy optimization principles and their applications. Springer; 1992.
3. Berger M. Geometry i. Springer Science & Business Media; 2009.
4. Asmussen S, Glynn PW. Stochastic simulation: algorithms and analysis. vol. 57. Springer Science & Business Media; 2007.
5. Spivak M. Calculus on manifolds. vol. 1. WA Benjamin New York; 1965.
