# Supplementary figures and images for: Uncertainty analysis of species distribution models
Source: PLoS One. 2019 May 23;14(5):e0214190. doi: 10.1371/journal.pone.0214190 (PMC6533036; doi:10.1371/journal.pone.0214190)

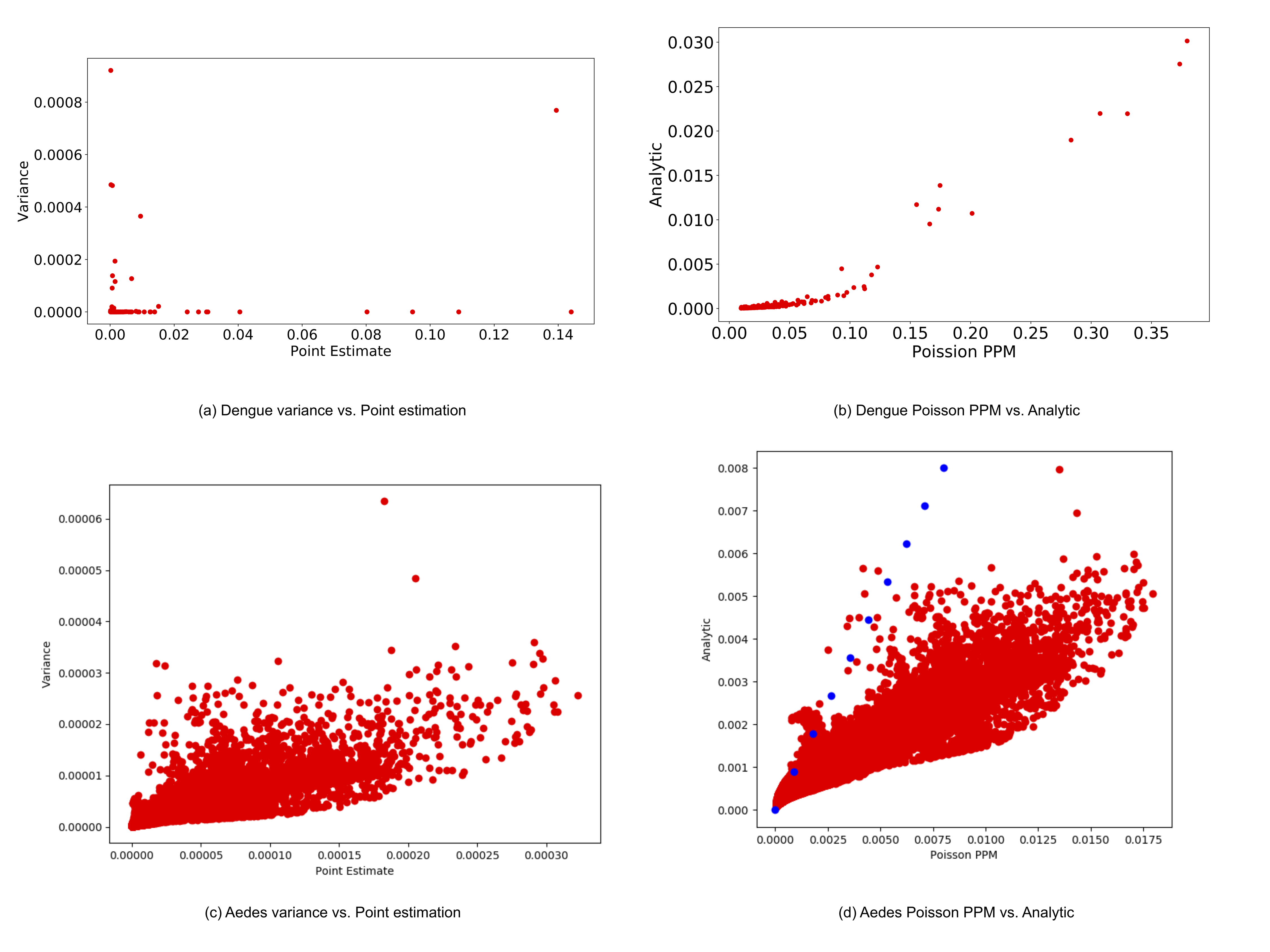

Supplement: S1 Figs — (a) Figure plots the relationship between point estimates of Dengue importation probability vs. variance calculated through analytic method. Non-linear relationship indicates the improper use of Poisson PPM for Dengue importation cases. (b) Figure plots the standard deviations of Poisson PPM vs. analytic for Dengue importation case study and indicates that Poisson PPM provides much larger standard deviation for Dengue imports application. (c) Figure plots the relationship between point estimates of Aedes Aegypti existence probability vs. variance calculated through analytic method. (d) Figure shows the standard deviation comparison between analytic method and Poisson PPM of Aedes Aegypti existence probability. (TIF) [file pone.0214190.s002.tif]
